# Novel Avian Coronavirus and Fulminating Disease in Guinea Fowl, France

**DOI:** 10.3201/eid2001.130774

**Published:** 2014-01

**Authors:** Etienne Liais, Guillaume Croville, Jérôme Mariette, Maxence Delverdier, Marie-Noëlle Lucas, Christophe Klopp, Jérôme Lluch, Cécile Donnadieu, James S. Guy, Léni Corrand, Mariette F. Ducatez, Jean-Luc Guérin

**Affiliations:** French National Institute for Agricultural Research (INRA), Toulouse, France (E. Liais, G. Croville, M. Delverdier, M.-N. Lucas, L. Corrand, M.F. Ducatez, J.-L. Guérin);; Ecole Nationale Vétérinaire, Toulouse (E. Liais, G. Croville, M. Delverdier, M.-N. Lucas, L. Corrand, M.F. Ducatez, J.-L. Guérin);; INRA 31326, Castanet-Tolosan, France (J. Mariette, C. Klopp; J. Lluch, C. Donnadieu);; North Carolina State University, Raleigh, North Carolina, USA (J. Guy)

**Keywords:** coronavirus, guinea fowl, metagenomics, next-generation sequencing, viruses, France, avian coronavirus, zoonoses, fulminating disease

## Abstract

For decades, French guinea fowl have been affected by fulminating enteritis of unclear origin. By using metagenomics, we identified a novel avian gammacoronavirus associated with this disease that is distantly related to turkey coronaviruses. Fatal respiratory diseases in humans have recently been caused by coronaviruses of animal origin.

Fulminating disease (also referred to as X disease) of guinea fowl (*Numida meleagris*) is an acute enteritis characterized by intense prostration and a very high death rate, leading to the almost complete destruction of affected flocks. Lesions are generally limited to severe enteritis and, in some birds, pancreatic degeneration. This disease is uncommon, but its fulminating evolution raises concerns of differential diagnoses with highly pathogenic avian influenza.

Fulminating disease has been reported for decades in the French guinea fowl industry, and although its viral origin was previously suspected, the virus remained unknown. During the 1980s, French groups investigated the etiology of the disease. Because propagation on cells or embryos remained unsuccessful and molecular tools were unavailable, etiologic hypotheses were based mostly on electron microscopy findings. The groups reached different conclusions, proposing candidates such as toga-like virus (*1*), reovirus, or herpesvirus (*2*). More recently, astroviruses have been associated with catarrhal enteritis in guinea poults (i.e., young guinea fowl) (*3*), but these viruses were not detected in birds affected by fulminating disease (data not shown).

We investigated field cases and performed an experimental reproduction of fulminating disease and identified its agent by using unbiased high-throughput sequencing. We propose a gammacoronavirus of a novel genotype as the most likely causal agent of fulminating disease. Coronaviruses (CoVs) are zoonotic. The novel human Middle East respiratory syndrome CoV, a betacoronavirus that was first isolated in 2012 in Saudi Arabia, is most closely related to *Tylonycteris* bat CoV HKU4 and *Pipistrellus* bat CoV HKU5 (*4*); severe acute respiratory syndrome–CoV originated from a betacoronavirus that spread from bats to civets and humans (*5*).

## The Study

We investigated field cases of fulminating disease among 5 flocks of guinea fowl in France during 2010 and 2011; in all cases, the birds’ clinical signs were limited to severe prostration, a dramatic decrease in water and feed consumption, and a daily death rate of up to 20%. From each affected flock, 5–10 sick birds were euthanized, and necropsy was performed. Most birds showed marked congestive enteritis and a whitish, enlarged pancreas. Livers, spleens, pancreas, kidneys, and intestinal tracts from the birds were placed into 10% buffered formalin for histopathologic examination or stored at −80°C for virologic analyses.

Fifteen 6-week-old guinea fowl poults were housed in 2 poultry isolators. One group of 5 birds that had been orally inoculated with the clarified and filtered (0.45-μm mesh) intestinal contents of diseased birds from 1 field case flock shared an isolator with another group of 5 uninoculated guinea poults, placed as contact birds. A third group of 5 uninoculated birds were placed in the other isolator. Inoculated and contact birds showed severe prostration as early as 2 days after infection and died or had to be euthanized within 6 days. Uninoculated birds showed no clinical signs of illness throughout the experiment. Necropsy was performed on birds that died or were euthanized, and comprehensive samples of tissues and fluids were obtained for subsequent investigations. At necropsy, the most notable lesion was an acute and marked enteritis; in some birds, a mildly enlarged and whitish pancreas was observed.

**Figure 1:**
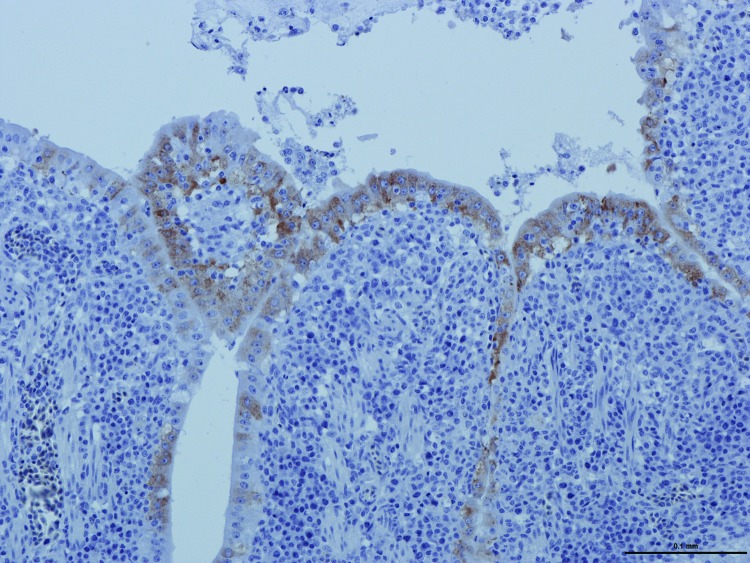
Replication of guinea fowl coronavirus in intestinal epithelium cells of experimentally infected birds as evidenced by immunohistochemical testing with a turkey coronavirus–specific monoclonal antibody, France, 2010–2011 (*9*). Scale bar indicates 0.1mm.

**Figure 2:**
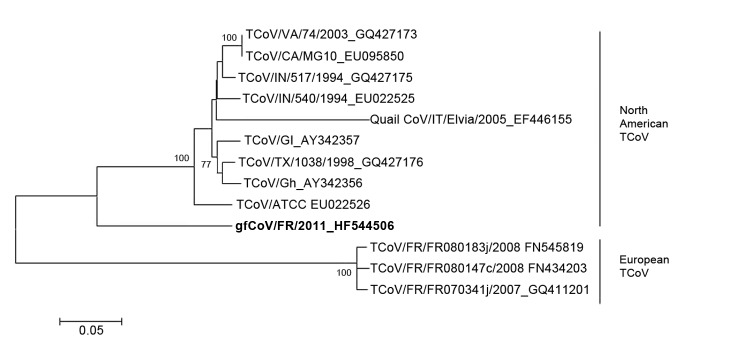
Phylogenetic analysis of the complete spike (S) gene of the fulminating disease of guinea fowl coronavirus (gfCoV, in **boldface**) in relation to turkey coronaviruses (TCoVs) at the nucleotide level. The tree was generated by using MEGA 5.05 (http://megasoftware.net/mega.php) by the neighbor-joining method, Kimura 2-parameter model, and pairwise deletion. Bootstrap values (1,000 replicates) >75 are indicated on the nodes. Only a partial S gene sequence (1,657 nt) was available for quail CoV/Italy/Elvia/2005. Scale bar indicates kimura distance.

The intestinal contents of experimentally infected poults were pooled, clarified by centrifugation, and pelleted by ultracentrifugation (100,000 × *g*, 2 h). The pellets were treated with RNase (Ambion, 20 µg/mL) and DNase (Invitrogen, 10 U/µL) (both from Life Technologies, Grand Island, NY, USA) to concentrate the viral material. RNA and DNA were extracted separately, and a random reverse transcription PCR was performed, as described (*6*), to generate unbiased PCR products of ≈300 bp. High-throughput sequencing was performed by using a MiSeq platform (Illumina, San Diego, CA, USA). A total of 476,430 sequences were generated (Table 1), 10.8% of which matched with known viral sequences, as determined by using GAAS software (http://gaas.sourceforge.net/) (*7*) with an expected value of 10^−3^; 7.5% of the eukaryotic viral reads were similar to avian CoVs, such as turkey CoV (TCoV) and infectious bronchitis virus (IBV). A total of 1,444 reads related to CoVs were aligned against the most similar TCoV genome available in GenBank (TCoV/CA/MG10; accession no. EU095850). The reads fairly aligned to almost the whole TCoV genome; the overall coverage was 78.86% (data not shown). A CoV-specific reverse transcription PCR was performed and the result was positive, specifically in intestinal tissues of experimentally infected birds (Table 2) (*8*). Furthermore, immunochemistry was performed by using a monoclonal antibody specific for TCoVs (*9*). We observed an intense, cytoplasmic, and granular labeling in enterocyte villi of inoculated birds only (Figure 1), suggesting a substantial intestinal replication of a TCoV-related virus. A 3,680-nt consensus full sequence of the spike (S) gene was completed by classical PCR and Sanger sequencing (GenBank/EMBL accession no. HF544506). A BLAST (http://blast.ncbi.nlm.nih.gov/Blast.cgi) search followed by a phylogenetic analysis performed on the complete S gene showed that guinea fowl fulminating enteritis virus corresponds to a distinct genotype of CoV, clustering within *Gammacoronavirus* genus, which also includes TCoV and IBV (*10*). The complete S gene sequence of guinea fowl CoV (GFCoV) was most similar to that of the TCoV S gene (minimum Kimura distance 22.6% at the nucleotide level between GFCoV/FR/2011 and TCoV/VA/74/2004, TCoV/CA/MG10, TCoV/IN/517/1994, or TCoV/ATCC). The GFCoV S gene was more similar to those of North American TCoV than to those of European (French) TCoV strains (Figure 2) (*11*).

**Table 1:** Distribution of reads generated by sequencing of pooled intestinal contents from guinea fowl poults with fulminating disease France, 2010–2011*

Read type	No. (%) reads
Total reads generated	476,430
Cellular reads	142,739 (30)
Bacterial reads	246,787 (51.8)
Archaea reads	35,271 (7.4)
Phage, plant, and insect virus reads	32,477 (6.8)
Eukaryote virus reads	19,155 (4.0)
Coronavirus reads	1,441 (7.5)†

*Sequencing was performed by using MiSeq (Illumina, San Diego, CA, USA) and analyzed by using the GAAS (http://gaas.sourceforge.net/) algorithm with an expected value of 10^−3^ (*7*). †Percent within eukaryote virus reads.

**Table 2:** Tissue tropism of coronavirus in experimentally and naturally infected guinea fowl poults, as detected by reverse transcription PCR, France, 2010–2011*

Case	Duodenum	Ileum/colon	Pancreas	Spleen	Bursa of Fabricius
Inoculated	5/5	5/5	0/5	0/5	1/5
Contact	3/5	5/5	0/5	0/5	1/5
Uninfected control	0/5	0/5	0/5	0/5	0/5
Field†	5/5	5/5	NT	NT	NT

*Data are no. of coronavirus PCR–positive birds/total no. of birds. NT, not tested. †Intestines (pooled duodenum, ileum, colon samples) from 5 field cases were tested (5–10 birds tested per clinical case). The 5 cases were selected on the basis of clinical signs (severe prostration and death rate >50%).

CoVs are positive-sense RNA viruses that are subject to frequent mutations and recombination events, resulting in the emergence of novel viruses, such as severe acute respiratory syndrome (in 2003) and Middle East respiratory syndrome CoV in humans (*4*,*5*). Avian CoVs associated with nonclinical carriage or with respiratory, genital, renal, or enteric diseases have been identified in many avian species (*12*). CoV infection causes mild enteritis in different avian species, mainly turkeys, partridges, and quails. Avian CoVs are usually classified as gammacoronaviruses, although a few bird CoVs have also recently been described as deltacoronaviruses (*13*). 

In the past, a recombination event led to the emergence of TCoV: the S gene of IBV recombined with an unknown virus (likely of avian origin), which resulted in a host change (chicken to turkey) and a tropism switch (respiratory to enteric). IBV and TCoV share <36% similarity for the S gene, but their full genomes are >86% similar (*14*). Although the origin of GFCoV is still unknown, the distance of its S gene to TCoV and IBV S genes suggests not only a common ancestor but also a current separate evolutionary path. A quail CoV similar to TCoV has been described (*15*); its available S gene sequence also clusters with North American TCoV (Figure 2). However, the comparable partial S gene sequences of quail CoV/Italy/Elvia/2005 and GFCoV differ greatly (genetic distance 30%). A few cases of guinea fowl fulminating disease are diagnosed each year in France but have no apparent epidemiologic link to each other. The severity of the disease in the field may suggest a poor adaptation of the pathogen to guinea fowl. This pathologic pattern differs greatly from TCoV enteritis in turkeys and makes GFCoV of potential interest for comparative studies of CoV pathobiology. Virus reemergence may indicate that >1 other species may also be asymptomatic carriers.

## Conclusion

We identified an avian gammacoronavirus related to TCoVs in fulminating disease of guinea fowl. The epidemiologic reservoir of this virus still needs to be clarified, and the sequencing of the full genome of the pathogen is warranted to fully assess its phylogenetic relationship with other gammacoronaviruses, its epidemiology, and its origin.
